# A rare case of simultaneous rectal and gastric carcinomas accompanied with inferior mesenteric arterioportal fistula: case report

**DOI:** 10.1186/s40792-019-0630-9

**Published:** 2019-05-17

**Authors:** Kengo Kai, Koichiro Sano, Kazuhiro Higuchi, Shuichiro Uchiyama, Hideto Sueta, Atsushi Nanashima

**Affiliations:** 1 Department of Surgery, Miyakonojo Medical Association Hospital, 1364-1 Tarobo, Miyakonojo, Miyazaki 885-0002 Japan; 2 Faculty of Medicine, Department of Surgery, University of Miyazaki, 5200 Kihara, Kiyotake, Miyazaki, Miyazaki Japan

**Keywords:** Inferior mesenteric arterioportal fistula, Rectal cancer, Gastric cancer, Low anterior resection, Interventional embolization, Portal hypertension

## Abstract

**Background:**

Inferior mesenteric arterioportal fistula (APF) is rare as only 35 case reports in the literature. We herein presented a case of simultaneously double cancer in the rectum and stomach with inferior mesenteric APF, which is the first case report by searching using PubMed. Combination of interventional embolization and surgical operation seemed to be optimal treatment for avoiding postoperative complications and the curability.

**Case presentation:**

A 66-year-old male with epigastric pain was admitted to a practitioner. He underwent a gastroscopy with biopsy, and cancer located in the lesser curvature of the gastric cardia was found. Enhanced CT did not reveal wall thickening of the stomach and distant metastases, but several swollen lymph nodes were observed in the right cardia. In the arterial phase, dilation of inferior mesenteric vein (IMV) and superior rectal artery (SRA) were noted, which raised suspicions of an arterioportal communication. Colonoscopy revealed a type 2 rectal tumor located 12 cm from the anal verge. The histological diagnosis of well-differentiated tubular adenocarcinoma was confirmed by biopsy. At a first step, we planned to perform a radiological embolization of inflow vessels to APFs except for SRA. Additionally, we determined the interval time of 1 month between the first low anterior rectal resection and the sequential gastrectomy for the purpose of decreasing portal pressure. The postoperative course was uneventful without hemorrhagic complications, and S-1 was taken internally 1 year as adjuvant chemotherapy for gastric cancer. The patient still lives without recurrence of this cancer with APF and portal vein thrombosis 2.5 years after the aforementioned surgeries.

**Conclusion:**

Inferior mesenteric APF and/or arteriovenous fistula (AVF) would be consisted of the several inflow arteries as superior rectal, internal iliac, and median sacral arteries, and outflow veins as inferior mesenteric, internal iliac, and median sacral veins. To determine the therapeutic strategy for left-sided colorectal cancers with abnormal vessel communications of the pelvis, it is significant to comprehend distribution and component vessels of APF and/or AVF.

## Background

Arterioportal fistula (APF) was firstly confirmed by Weigert [1] in 1886. Recently, the number of reported cases of APF has increased with developments in imaging modalities including multi-detector computed tomography (CT) or selective angiography. APF can be observed in variable locations. Detection is mostly developed in the celiac artery or its branches, notably the hepatic (45%) or the splenic (30%) artery [2]. However, APF involving the inferior mesenteric vessels is uncommon, and only 35 cases of inferior mesenteric APF have been reported thus far [2–36]. In this report, we present a case with simultaneous double cancers in the rectum and stomach accompanied by inferior mesenteric APF, which could be successfully treated by a combination of interventional embolization and surgical excision. To our knowledge, there have not been any reports similar to the present one. Finally, we reviewed the course of treatment and the accompanying literature, which should help clinicians encountering similar cases.

## Case presentation

A 66-year-old male with epigastric pain was admitted to a practitioner. He underwent a gastroscopy with biopsy, and cancer located in the lesser curvature of the gastric cardia was found. The patient was transferred to our hospital for surgical treatment. He was in good health except for mild obesity (body mass index of 27) and a history of appendectomy. Abdominal examination revealed tenderness in the epigastric fossa. However, no palpable pulsatile mass was confirmed. There was no symptom of hypertension and heart failure. Laboratory findings were unremarkable except for high hemoglobin A1c levels as 6.7% (ranging 0–6%). Ultrasonic cardiography showed no remarkable right heart load and pulmonary hypertension.

Upper gastrointestinal endoscopy showed an irregular ulcerated lesion with fold convergence, encroachment, and poor dispensability in the lesser curvature of the cardia, which was diagnosed as submucosal or muscular proprial infiltration by gastric cancer. Histological diagnosis revealed a moderately-to-poorly differentiated adenocarcinoma. Neither varices nor portal hypertensive gastropathy was remarkable.

**Fig. 1:**
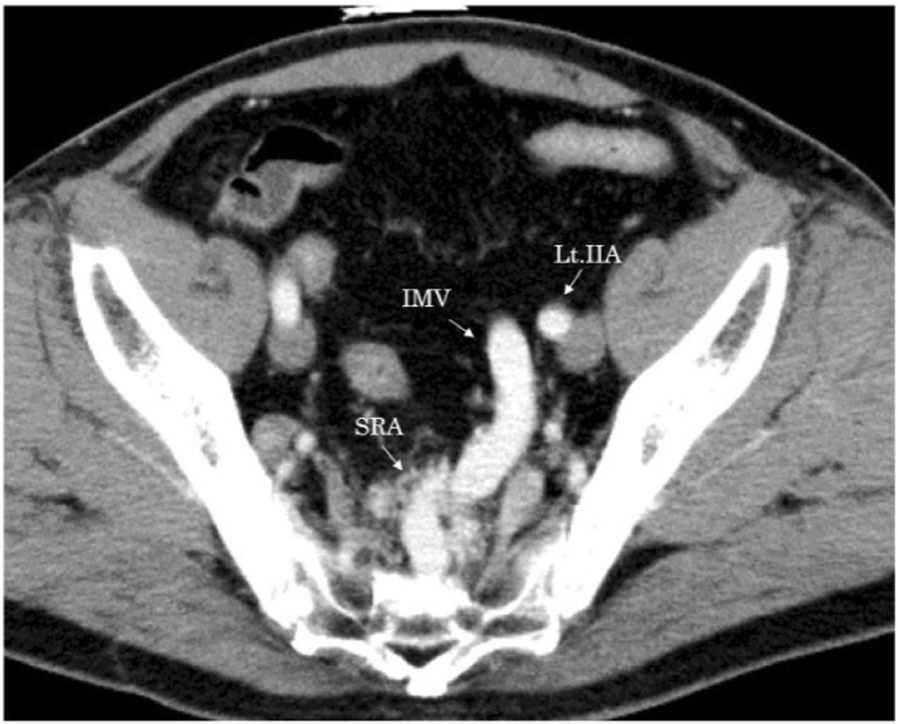
Enhanced computed tomography showed dilation of inferior mesenteric vein (IMV) and superior rectal artery (SRA) was noted in arterial phase. Lt IIA: left internal iliac artery

**Fig. 2:**
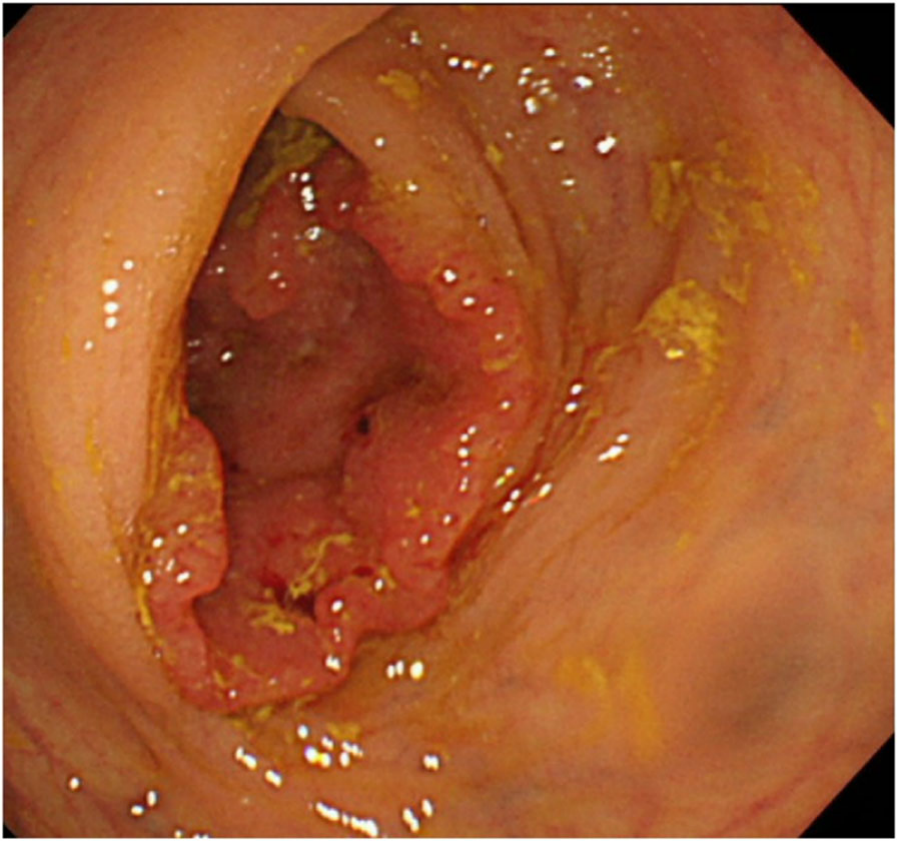
Colonoscopy revealed type 2 rectal tumor located 12 cm from the anal verge. Background mucosa did not show edematous changes or ulcerative and hemorrhagic lesions

Enhanced CT did not reveal wall thickening of the stomach and distant metastases, but several swollen lymph nodes were observed in the right cardia. In the arterial phase, dilation of the inferior mesenteric vein (IMV) and superior rectal artery (SRA) were noted, which raised suspicions of an arterioportal communication (e.g., a fistula between the branches of the inferior mesenteric artery (IMA) and the SRA (Fig. 1)). Furthermore, wall thickening with enhancement of rectum was observed, which was hypothesized to be an intestinal edema due to APFs or rectal cancer. Colonoscopy revealed a type 2 rectal tumor located 12 cm from the anal verge. Background mucosa did not show edematous change or ulcerative and hemorrhagic lesions (Fig. 2). The histological diagnosis of well-differentiated tubular adenocarcinoma was confirmed by biopsy.

**Fig. 3:**
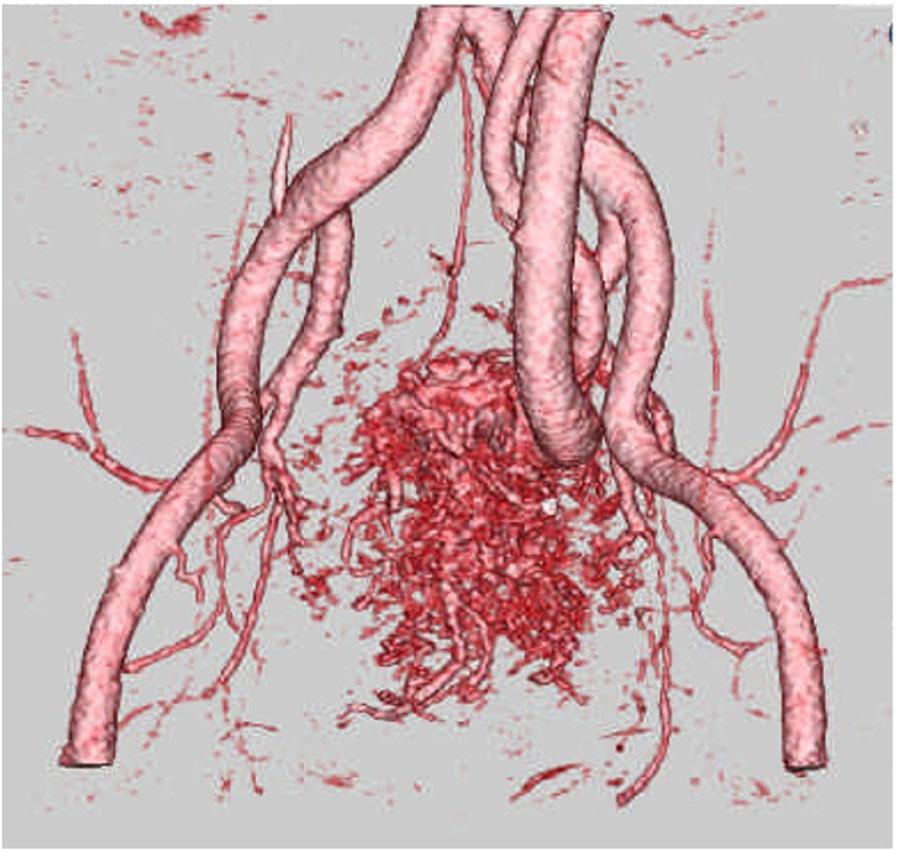
Four-dimensional CT angiography demonstrated that tortuous and dilated IMV supplied blood flow through many fistulas from superior mesenteric artery, bilateral internal iliac artery, and median sacral artery

**Fig. 4:**
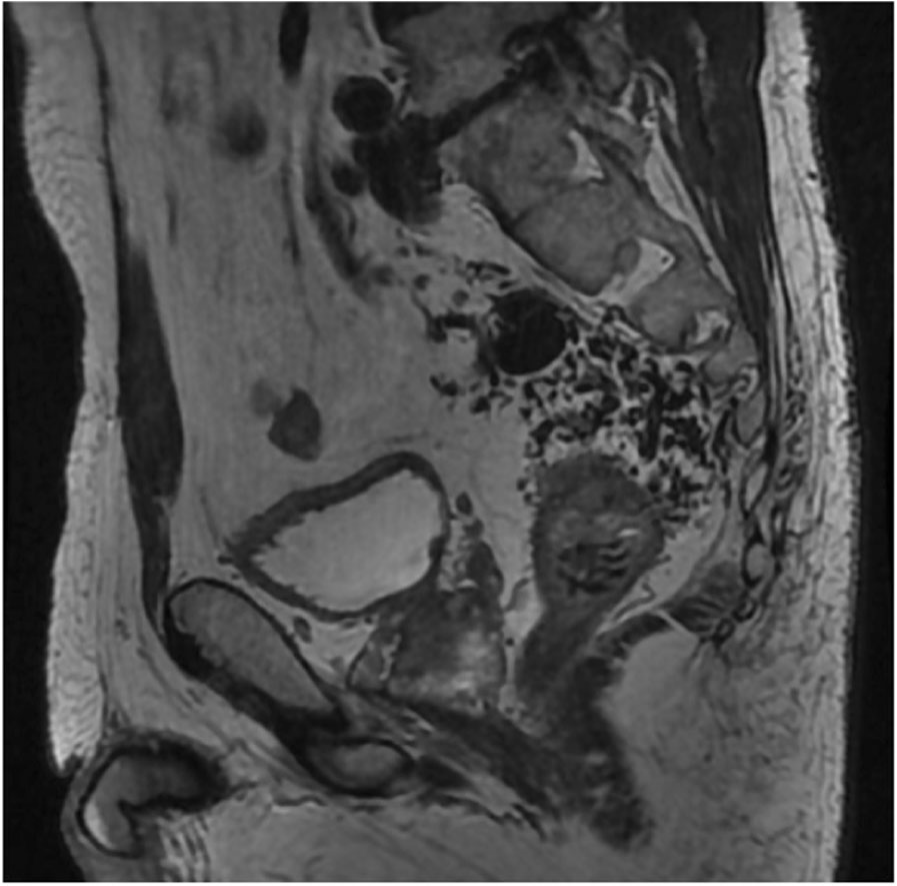
Abdominal enhanced magnetic resonance imaging revealed blood inflow from branches of the median sacral artery to APFs by flow void at the pelvic surface of the sacrum

**Fig. 5:**
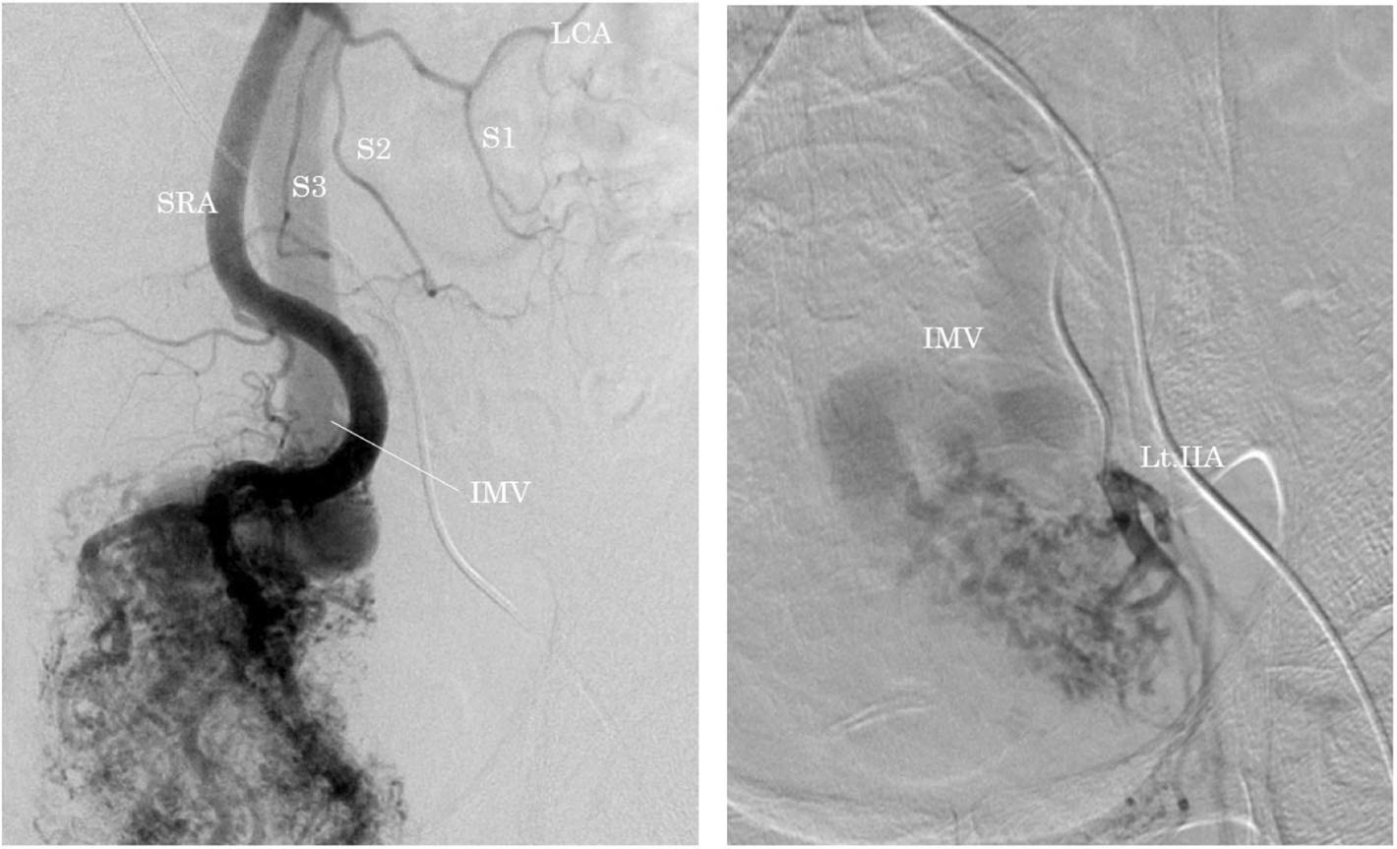
Selective angiography showed that the superior rectal artery (SRA) occupied more blood inflow to APFs in comparison with the bilateral internal iliac artery (IIA) and median sacral artery. IMV, inferior mesenteric vein; LCA, left colic artery; S1, 1st sigmoid artery; S2, 2nd sigmoid artery; S3 3rd sigmoid artery

Four-dimensional CT angiography demonstrated that the tortuous and dilated IMV supplied blood flow through several fistulas including the SRA, bilateral internal iliac artery (IIA), and median sacral artery (Fig. 3). Abdominal enhanced magnetic resonance imaging (MRI) revealed blood inflow from branches of median sacral artery to APFs by flow void at the pelvic surface of sacrum (Fig. 4), which did not identify a nidus, an abnormal vascular mass with an arteriovenous malformation. Selective angiography showed that SRA occupied more blood inflow to APFs in comparison with the bilateral IIA and median sacral artery (Fig. 5). The superior mesenteric artery did not contribute to the constitution of the APFs.

These abnormal vessel communications were diagnosed as APFs which used the SRA, bilateral IIA, and median sacral artery as inflow vessels and the IMV as outflow vessel. Additionally, the synchronous double cancer was diagnosed as gastric cancer (U, Less, cT2, cN1, cM0, cStageIIA) and rectal cancer (Ra, cT3, cN0, cM0, cStageII) using the Union for International Cancer Control’s classification [37]. We were aware of intraoperative bleeding complications due to many vessels penetrating the mesorectum from the IIA and median sacral artery in a low anterior resection. Furthermore, it was possible that occult portal hypertension due to dilated IMV resulted in a hemorrhagic tendency in gastrectomy. At a first step, we planned to perform a radiological embolization of inflow vessels to APFs except for SRA. Additionally, we determined the interval time of 1 month between the first low anterior rectal resection and the sequential gastrectomy for the purpose of decreasing portal pressure.

**Fig. 6:**
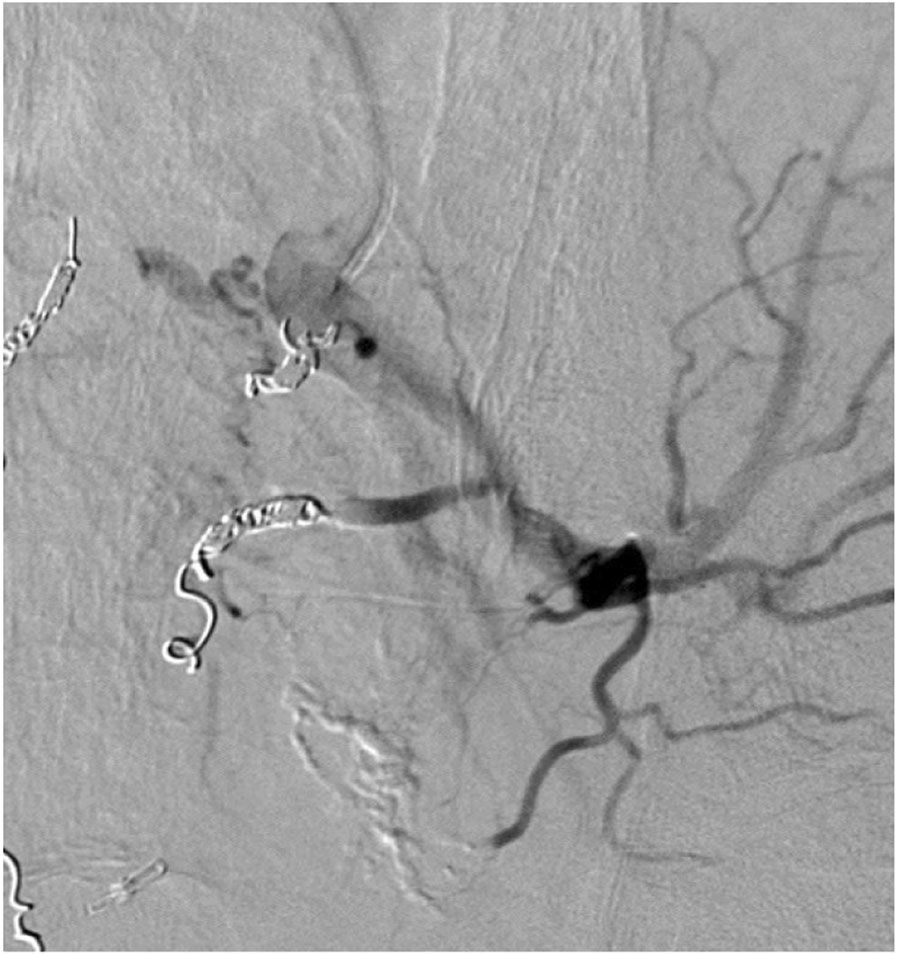
Fistula embolization was performed for the branches of the median sacral artery and bilateral internal iliac artery with micro coil, gelatin sponge, and n-butyl-2-cyanoacrylate

**Fig. 7:**
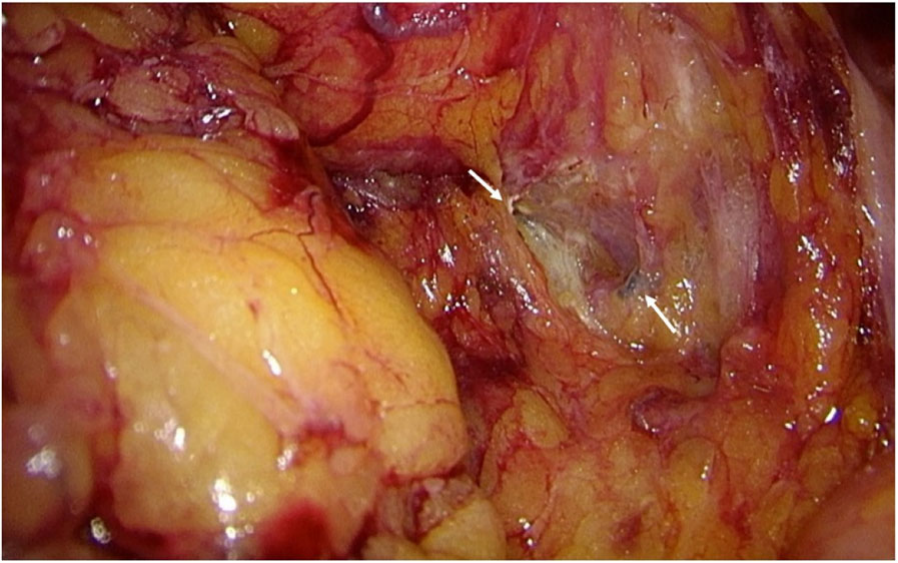
Laparoscopic low anterior resection was performed on the day following IVR. Coils in inflow vessels were revealed on the exfoliated surface in the pelvis

Fistula embolization was performed for the branches of the median sacral artery and bilateral IIA using a micro coil, gelatin sponge, and n-butyl-2-cyanoacrylate (Fig. 6). Post-treatment aortography revealed hypo-perfusion of APFs. On the day following interventional radiology (IVR), the patient underwent laparoscopic low anterior resection with D3 lymphadenectomy. We reasonably assumed that the fistula was located in mesorectum because the coil was exposed to inflow vessels on the exfoliated surface of the pelvis (Fig. 7). Intestinal reconstruction was anastomosed with the double stapling technique without additional covering colostomy. Intraoperative blood loss was 165 g. The freshly resected specimens revealed a 68 × 38 mm of type 2 tumor without erosions or ulcer on the mucosa. Pathological findings diagnosed the rectal cancer (Ra, Type2, pT3, pN0 (0/14), pStageII) [37].

**Fig. 8:**
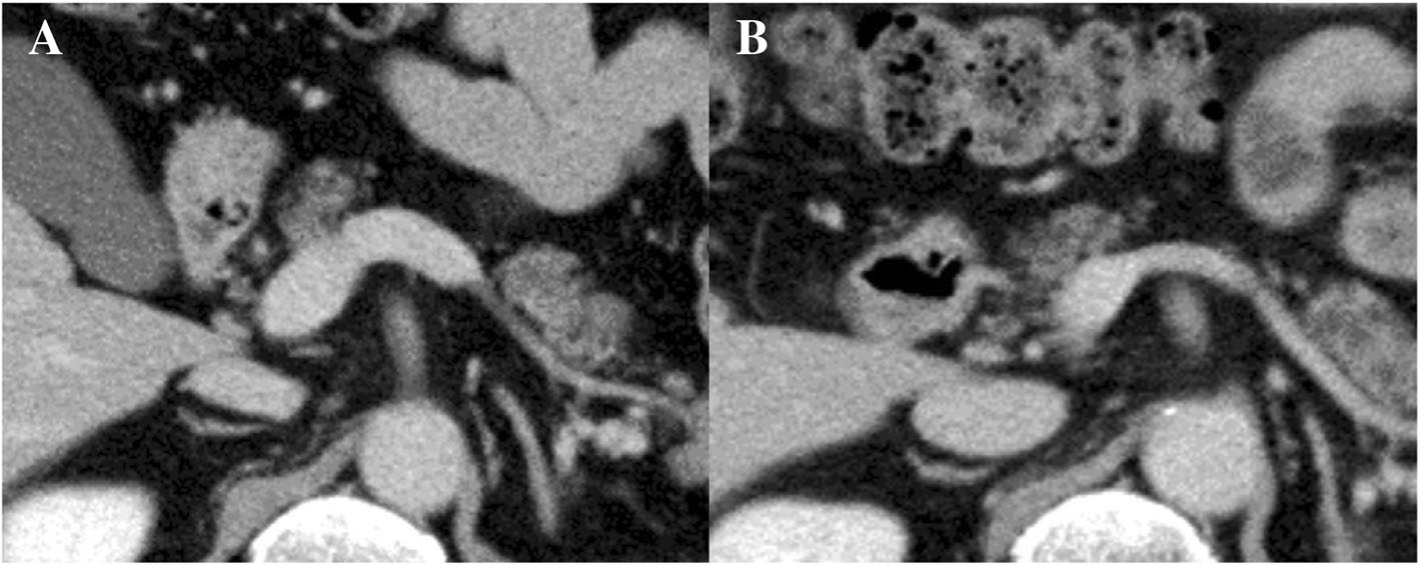
Abdominal CT taken on the postoperative day 28 (**b**) showed that the diameter of the portal vein was diminished in size compared to its preoperative state (**a**)

Abdominal CT performed on postoperative day 14 showed a small fluid collection with gas around the anastomosis, which suggested an anastomotic leakage and thrombosis, which emerged in the right branch of the portal vein and remnant IMV. After intake of warfarin for 2 weeks, thrombotic size was markedly decreased, and the diameter of portal vein diminished in size compared to its preoperative state (Fig. 8). The 1-week fasting cured the anastomosis leakage without generating a stoma. The patient underwent total gastrectomy with a Roux-en-Y reconstruction 1 month after surgery for rectal cancer. No over-swelling of portal vein system or splenectasis was found, and blood loss was 410 g. The final gastric cancer staging was U, Less, Type3, pT3, pN1a(2/37), pStageIIB [37].

The postoperative course was uneventful without hemorrhagic complications, and S-1 was taken internally 1 year as adjuvant chemotherapy for gastric cancer. The patient still lives without recurrence of this cancer with APF and portal vein thrombosis 2.5 years after the aforementioned surgeries.

## Discussion

APF involving the IMV is extremely rare, and to our knowledge, only 35 cases have been reported in the literature to date [2–36]. Arteriovenous fistula (AVF) and arteriovenous malformation (AVM) have similar vascular pathogeneses. In one study [38], AVM was defined as having an abnormal aneurysm called a “nidus”. In contrast, AVF or APF is considered to communicate inflow arteries and outflow veins, or portal veins in main or segmental branches without a nidus. However, there is confusion because there are no clear criteria for classification, and diagnosis with fresh specimen is difficult pathologically. In this report, we discuss APF as an abnormal vessel communication, in which IMV acts the major outflow vessel regardless of the existence of a nidus.

APFs usually occur secondarily to previous surgeries, trauma, neoplasms, and rupture of an arterial aneurysm into the adjacent vein [14]. Other APFs arise from congenital disease such as AVM and Osler-Rendu-Weber disease [13, 15, 21]. Iatrogenic APF is thought to be caused by vessel injury due to mesenteric hematoma or infection in unclean surgery. The present case is an idiopathic occurrence without any of the previous history described above, and its etiology was therefore unknown.

The presenting symptom in the majority of cases included abdominal pain and mass. It has been hypothesized that increased blood flow through APF causes portal venous hypertension, and a decreased arterial flow to the bowel wall results in non-occlusive ischemic colitis secondary to steal phenomenon. Clinical sign and symptoms were attributed to either portal hypertension including splenomegaly, ascites, and bleeding esophageal varices or ischemic colitis, which included diarrhea and lower gastrointestinal bleeding. In this patient, although no symptoms of portal hypertension and ischemic colitis were observed in the preoperative assessment, the reduced size of the portal vein detected in the postoperative CT demonstrated occult portal hypertension. Portal hypertension accompanies 42% of APF cases due to an increase of outflow and intrahepatic vascular resistance [4]. Adamsons [39] noted that arterialization of the portal vein caused sclerosis of the portal venous radicles, which increased resistance to portal venous inflow. The induced fibrosis may induce a perpetuated portal hypertension even when portal flow is restored in the normal range. Their study suggested that APF produces irreversible changes in the liver. Fuji et al. [38] argued that all APF with or without symptoms could be treated at the earlier stage when it is detected. However, 4.5% of APF cases may have symptoms of heart failure with systematic effects [13].

Direct angiography is the most important modality for the diagnosis and crafting strategy for APF [4]. Inferior APF consists not only of the inferior mesenteric artery and vein but also complex vessels such as the internal iliac, inferior mesenteric, and median sacral artery and vein. The differential diagnosis of inferior APF of the pelvic AVM in the internal iliac perfusion regions is often difficult [40, 41]. Angiography is essential to assess every blood flow and the presence of nidus and vascular fistulas. Recently, instead of the direct angiography, MR angiography and 4D-CT have been useful as a non-invasive investigation tool to define cryptogenic portal venous hypertension or ischemic colitis [4].

The treatment of APF is mainly performed by the intra-arterial embolization, surgery, and these combined procedures, and the approximately 90% of AVM cases are treated regardless of embolization or surgical resection [2]. The embolization procedure is an effective management technique, and surgical resection can be avoided if selected. Embolization is less invasive and relatively safe in comparison with surgical procedures. However, there is a risk of an associated organ ischemia or the fistula recurrence in cases when more than one feeding vessel is involved. Furthermore, the migration of embolization material resulted in ischemic colitis or pulmonary embolism, which is likely when the APF diameter is over 8 mm and the flow rate is high [8]. In such a situation, according to some case reports [35], the construction of covering a stoma on colectomy and anastomosis is necessary. Thus, ischemia or edema arisen by resection of APF is a concern. We inosculated without stoma because preoperative colonoscopy showed no sign of blood circulation failure, and radiological selective embolization to the branch of main arteries could preserve the flow to the colonic wall.

In this case, we could not assess the existence of APFs in mesorectum with pathological examination, because of taking priority over assessment for dissected lymph nodes, regrettably. We guessed that APFs was located in the mesorectum because postoperative CT did not show APFs.

We considered that median sacral artery flowed into APFs without going through middle rectal artery from the findings of flow void in the preoperative MRI. On the other hand, the possibility that bilateral IIA contributed to the constitution of APFs via middle rectal artery cannot be denied. So, we cannot affirm the validity and efficiency of bilateral IIA embolization in this case, as compared to sacral artery embolization.

As described above, our present case is extremely rare because it entailed a double cancer with the inferior mesenteric APF. We were eventually able to treat it without bleeding complication by preceding IVR for APF. Two cases in the left-side colorectal cancer with comorbid abnormal vessel communications in pelvis are reported in Table 1 [40, 41]. However, both clearly differentiated from our case because the nidi were located in internal iliac region, and main in- and outflow vessels were related to systemic circulation. These patients had asymptomatic pelvic AVM diagnosed preoperatively for colorectal cancers and were observed without treatment for AVM because of concerns about the risk of heart failure.

**Table 1:** Cases of left-sided colorectal cancer comorbid abnormal vessel communications of pelvis

Case	Sex	Age	Location of cancer	Clinical symptom	Location of APF	Main inflow vessel	Main outflow vessel	HF	PH	IVR	Operation for cancer
Honma et al 2010 [40]	M	60	S	-	Right IIA region	IIA	IIV	+	-	-	Partial resection of sigmoid colon
Takeda et al 2017 [41]	M	58	RS	-	Right IIA region	IIA	IIV	-	-	-	Laparoscopic high anterior resection
Our case	M	66	Ra	-	Mesorectum	SRA	IMV	-	+	+	Laparoscopic low anterior resection

*S* sigmoid, *RS* recto-sigmoid, *Ra* rectum above the peritoneal reflection, *HF* Heart failure, *PH* Portal hypertension, *IVR* Interventional radiological treatment, *IIA* Internal iliac artery, *IIV* Internal iliac vein, *SRA* Superior rectal artery, *IMV* Inferior mesenteric vei

## Conclusions

We reported a rare case of simultaneous rectal and gastric carcinomas accompanied with inferior mesenteric arterioportal fistula. To determine therapeutic strategies for left-sided colorectal cancer with abnormal vessel communications of the pelvis, it is vital to understand the distribution and component vessels of inferior APF in order to make proper decisions regarding preoperative APF treatments.

## Abbreviations

APF: Arterioportal fistula
AVF: Arteriovenous fistula
CT: Computed tomography
IIA: Internal iliac artery
IMA: Inferior mesenteric artery
IMV: Inferior mesenteric vein
IVR: Interventional radiology
SRA: Superior rectal artery
